# Olfactory Stimulation with Volatile Aroma Compounds of Basil (*Ocimum basilicum* L.) Essential Oil and Linalool Ameliorates White Fat Accumulation and Dyslipidemia in Chronically Stressed Rats

**DOI:** 10.3390/nu14091822

**Published:** 2022-04-27

**Authors:** Da-Som Kim, Seong-Jun Hong, Sojeong Yoon, Seong-Min Jo, Hyangyeon Jeong, Moon-Yeon Youn, Young-Jun Kim, Jae-Kyeom Kim, Eui-Cheol Shin

**Affiliations:** 1 Department of Food Science, Gyeongsang National University, Jinju 52725, Korea; kim94dasom@naver.com (D.-S.K.); ringspot@naver.com (M.-Y.Y.); 2 Department of GreenBio Science, Gyeongsang National University, Jinju 52725, Korea; 01028287383a@gmail.com (S.-J.H.); dbsthwjd0126@naver.com (S.Y.); jojo9875@naver.com (S.-M.J.); giddus9967@naver.com (H.J.); 3 Department of Food and Biotechnology, Korea University, Sejong 30019, Korea; yk46@korea.ac.kr; 4 Department of Behavioral Health and Nutrition, University of Delaware, Newark, DE 19716, USA; jkkim@udel.edu; 5 Division of Food Science and Technology, Agri-Food Bio Convergence Institute, Gyeongsang National University, Jinju 52725, Korea

**Keywords:** *Ocimum basilicum* L., essential oil, volatile compounds, linalool, stress lipid metabolism

## Abstract

We explored the physiological effects of inhaling basil essential oil (BEO) and/or linalool and identified odor-active aroma compounds in BEO using gas chromatography/mass spectrometry (GC–MS) and GC–olfactometry (GC–O). Linalool was identified as the major volatile compound in BEO. Three groups of rats were administered BEO and linalool via inhalation, while rats in the control group were not. Inhalation of BEO for 20 min only reduced the total weight gain (190.67 ± 2.52 g) and increased the forced swimming time (47.33 ± 14.84 s) compared with the control group (219.67 ± 2.08 g, 8.33 ± 5.13 s). Inhalation of BEO for 5 min (392 ± 21 beats/min) only reduced the pulse compared with the control group (420 ± 19 beats/min). Inhalation of linalool only reduced the weight of white adipose tissue (5.75 ± 0.61 g). The levels of stress-related hormones were not significantly different among the groups. The total cholesterol and triglyceride levels decreased after inhalation of BEO for 20 min (by more than −10% and −15%, respectively). Low-density lipoprotein cholesterol levels were lowered (by more than −10%) by the inhalation of BEO and linalool, regardless of the inhalation time. In particular, BEO inhalation for 20 min was associated with the lowest level of low-density lipoprotein cholesterol (53.94 ± 2.72 mg/dL). High-density lipoprotein cholesterol levels increased after inhalation of BEO (by more than +15%). The atherogenic index and cardiac risk factors were suppressed by BEO inhalation. Animals exposed to BEO and linalool had no significant differences in hepatotoxicity. These data suggest that the inhalation of BEO and linalool may ameliorate cardiovascular and lipid dysfunctions. These effects should be explored further for clinical applications.

## 1. Introduction

Stress is classified as either acute or chronic and can influence the physiological regulation of hormones and inflammatory cytokine secretion through several pathways, involving psychological, social, physical, and chemical factors [1,2,3]. Chronic stress usually disturbs the autonomic nervous system (ANS), which maintains internal homeostasis responding to changes in the external environment and controls the metabolism of substances in the body. Furthermore, the ANS generally regulates the sympathetic and parasympathetic systems, and thus, chronic stress can interfere with the activation of the sympathetic and parasympathetic systems [4]. The deterioration of the ANS usually increases blood pressure, pulse, total cholesterol level, and low-density lipoprotein cholesterol (LDL) levels and decreases high-density lipoprotein cholesterol (HDL) levels. Accordingly, pathologies of the ANS can induce a deterioration in cardiovascular health, leading to hypertension and arteriosclerosis [4,5]. Therefore, researchers have attempted to improve the cholesterol levels and prevent the progression of cardiovascular diseases using natural products with physiological effects [6].

Basil (*Ocimum basilicum Licorice; O. basilicum* L.) is a member of the *Lamiaceae* family. The leaf and stem parts, are used as culinary ingredients and/or as medicinal herbs [7]. Additionally, basil contains a unique fragrance that has been used in the perfume industry. Furthermore, the intake of basil has beneficial effects on the cholesterol level; the intake of basil improves lipid metabolism in high-cholesterol-affected animal models [7]. In addition, orally administered linalool, one of the major compounds in basil, improves cholesterol levels and, when administered by inhalation, induces sedative and relaxing effects [6,8]. Generally, the volatile profiles of essential oil are affected by many factors, such as the geographical area of sampling [9], the variety of/accession to the plants [10], the harvest year [11], the harvest date [12], the extraction system [13], so on. Therefore, the major aroma compound (linalool) of basil is mainly affected by geographical conditions and the harvesting periods, and the concentration of linalool increases according to the flowering periods [14]. In addition, linalool concentration is also affected by the extraction method. In particular, the hydro-distillation extraction method (18.1%) yields a higher concentration of linalool than supercritical fluid extraction (12.6%) [15].

When fragrant products are inhaled, individual fragrance compounds bind to nasal olfactory receptors, and a signal is transmitted to the cerebrum. When a volatile compound is inhaled, it dissolves in the mucus of the nasal mucous membrane and moves to the olfactory epithelium. Subsequently, volatile compounds bind to the olfactory receptors of cilia. Olfactory receptors bind only to certain volatile compounds, and the generated electrical signal reaches the olfactory bulb in the frontal lobe via axons. Therefore, information on individual volatile compounds is delivered to the olfactory cortex and the cerebrum. Individual volatile compounds can be distinguished and recognized according to this signaling pathway [16]. Olfactory stimulation influences the central nervous system (CNS) and ANS activities; thus, olfactory stimulation can control the function of the sympathetic and parasympathetic nervous systems. The involved nerves generally influence energy and lipid metabolism; thus, food intake and cholesterol levels can be controlled by the sympathetic and parasympathetic nervous systems [5,17].

Improvements in lipid metabolism in vivo by the intake of basil and sedative and anti-stress effects of linalool contained in basil have been reported [6,7,8]. However, the ameliorating effects of inhaling volatile compounds (present in basil) on dyslipidemia caused by chronic stress have not been elucidated. Accordingly, this study observed changes in lipid parameters, stress hormone levels, pulse, body weight, and food intake after inhalation of basil essential oil (BEO) in chronically stressed rats. Furthermore, changes in metabolic parameters following linalool inhalation were observed.

## 2. Materials and Methods

### 2.1. Essential Oil

The basil used in this study was cultivated in Austria in 2017, and the essential oil was extracted by the distillation method using the leaves (100%). The BEO used in this study was a commercial product, purchased from the Aroma Care Solution (Helga-Stolz GmbH Co., Grafenwoerth, Austria) and stored at 4 °C in a dark place until experiments were performed. The grade of this product was for aroma therapy. The experiments in the present study were conducted in 2018–2020.

### 2.2. Odor-Active Aroma Compounds

Odor-active aroma compounds (OAACs) in BEO were collected using solid-phase microextraction (SPME) fibers (Supelco Co., Bellafonte, PA, USA), i.e., fibers coated with 100 μm of polydimethylsiloxane (1 cm in size). BEO (1 g) was placed in a glass vial tightly sealed with an aluminum cap. The OAACs were collected in the headspace while heating the sample to 50 °C. The SPME fibers were injected into the injector of a gas chromatography–mass spectrometry selective detector (GC–MS; Agilent 7890A & 5975C, Agilent Technologies, Santa Clara, CA, USA) at 220 °C, and the analysis was performed after desorption for 10 min. The column was HP-5MS (30 m (length) × 0.25 mm (inner diameter), 0.25 μm (film thickness)), and helium carrier gas was used at 1 mL/min, with a split ratio of 1:10. The initial oven temperature was set at 40 °C for 5 min, increased to 200 °C at a rate of 5 °C/min, and maintained for 10 min. An inlet temperature of 220 °C was set in the splitless mode. OAACs, separated by a total ionization chromatogram, were identified using the National Institute of Standards and Technology (NIST) mass spectral library (NIST version 12). Pentadecane (0.005 µg) was used as an internal standard. According to the peak area and concentration of the internal standard, the concentrations of the OAACs in BEO were expressed in µg/mL. To explore the odor-active characteristics of BEO, the volatile profiles were separated by the GC column and assessed using a GC–olfactometry port (GC-O) (ODP 3, Gerstel Co., Linthicum, MD, USA). Odor-active intensity was divided into four levels, with higher levels representing stronger odor-active intensity, as described previously [5].

### 2.3. Animal Care and Experimental Design

**Figure 1:**
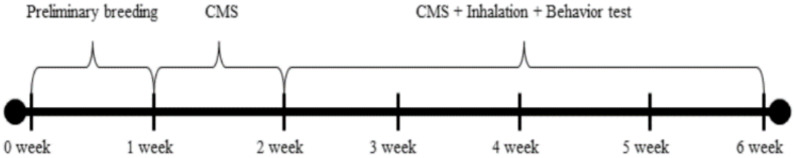
The plan of the animal study showing a week of preliminary breeding, a week of chronic mild stress (CMS), and four weeks of CMS + inhalation + behavior testing.

This study was approved by the Animal Experimental Ethics Committee (Animal protocol #: IACUC-4). Forty-five male Sprague–Dawley rats (4 weeks old) were obtained from Coretec Co., (Busan, Korea). The rats were acclimated to a normal diet for a week and randomly classified into four groups. After classification, chronic stress was applied to all groups for five weeks in total. Chronic mild stress was applied in the first week. Chronic mild stress (CMS) is a complex stress that includes food deprivation, restricted access to food, water deprivation, roommate separation, overnight illumination, and tilting the cage by 45°. From the second week, the rats were exposed to chronic stress with distilled water (DW) inhalation for 5 min/day in the control group (CON; *n* = 6), chronic stress with linalool inhalation for 5 min/day in the positive control group (POS; *n* = 6), chronic stress with BEO inhalation for 5 min/day in the third group (5 MIN; *n* = 6), and chronic stress with BEO inhalation for 20 min/day in the fourth group (20 MIN; *n* = 6) (Figure 1). Linalool and volatile compounds in BEO flowed at a rate of 8 mL/h, achieved by using a humidifier (Aroma diffuser humidifier; Cactus Co., Shanghai, China).

Food intake and body weight were measured once weekly. The rats were fasted for 16 h before dissection. Blood was collected from the heart using syringes containing 20 mg of ethylenediaminetetraacetic acid. The collected blood samples were kept for 30 min, then centrifuged at 1000 G to separate the serum. Finally, organs and tissues (the liver, kidneys, heart, white adipose tissue, and brown adipose tissue) were extracted and weighed. In addition, the organs and tissues were stored in a −80 °C freezer [5].

### 2.4. Forced Swimming Test

The forced swimming test was performed weekly with a standard behavioral despair test. Water (25 °C) was placed in a chamber (40 × 25 × 26.5 cm) at a height of 16 cm. During each experiment, the animals were placed in the chamber and allowed to swim (mobility). Immobility was assessed after swimming. Immobility was defined as when the animals stood upright and floated without movement, exposing only the head [18].

### 2.5. Pulse

The animal’s pulse was measured using the tail-cuff method with BP-2000 (Visitech Systems Co., Apex, NC, USA). Eight measurements were taken, excluding the highest and lowest values and deviations. Finally, three measured values were expressed as the average and standard deviation (SD) [5].

### 2.6. Stress Hormones

Cortisol (450 nm) in the serum was analyzed using an ELISA kit (YH ELISA Kit, Shanghai Yehua Biological Technology Co., Shanghai, China), and serotonin (450 nm) in brain tissue was analyzed using another ELISA kit (Serotonin ELISA Kit, Bio Vision Co., Milpitas, CA, USA) by absorbance measurement according to the manufacturer’s instructions [19,20].

### 2.7. Analyses of Serum Biomarkers and Hepatotoxicity

Total cholesterol (500 nm), HDL (500 nm), triglyceride (TG) (550 nm), and hepatotoxicity, including aspartate transaminase (AST) (505 nm) and alanine transaminase (ALT) (505 nm), were analyzed using a commercial kit (Asan Reagents, Asan Pharm Co., Seoul, Korea) by absorbance measurement according to the manufacturer’s instructions [5].

### 2.8. Statistical Analysis

Experiments were performed in triplicate, and the results are presented as the average and SD. Non-parametric comparison was used to compare paired groups using the Friedman test with chi-square distribution. Differences were considered statistically significant at *p*-values less than 0.05 (SAS Institute Inc., Cary, NC, USA).

## 3. Results and Discussion

### 3.1. Odor-Active Aroma Compounds

**Figure 2:**
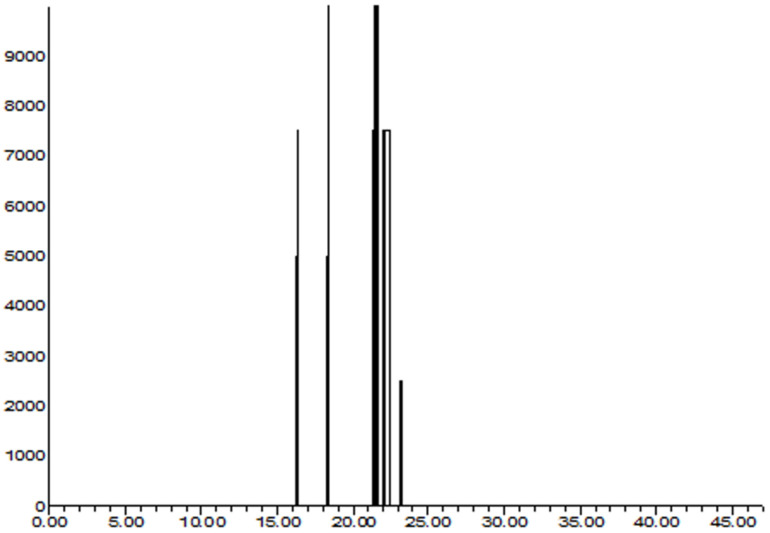
Representative aromagram of odor-active aroma compounds (OAACs) in basil (*Ocimum basilicum* L.) essential oil identified by GC–MS and GC–O test.

Odor-active aroma profiles were detected using GC–MS and GC–O (Table 1 and Figure 2). A total of 17 aroma compounds were detected in BEO. In particular, four OAACs were identified, including linalool, linalool oxide, menthane, and carvone. Linalool elicits basil essential oil odor activation, and linalool oxide elicits the activation of grass and herb odors. Menthane also elicits herb odor and menthol activation. In addition, carvone elicits lemon odor activation.

Linalool had the highest concentration of OAAC in BEO (Table 1). Linalool is a common and major terpenoid, containing most herbal essential oils and has been identified as a forest-like odor using GC–O [5,21]. In addition, linalool can control the lipid metabolism in vivo [21]. Linalool oxide has shown anxiolytic-like effects in mouse anxiety models via inhalation [22]. Menthene is a hydrocarbon with colorless characteristics and an herb odor [23]. D-Carvone has anti-inflammatory and anti-microbial effects, and this volatile compound was identified as OAAC in essential oils by GC–O [5,24]. In general, the genus *Ocimum* includes approximately 150 species distributed worldwide, and different volatile profiles are characteristic of *Ocimum* species, as reported by a previous study. Importantly, high concentrations of linalool were detected in O. *basilicum* L. (25.6%) and O. *sanctum* L. (21.9%) but not in O. *gratissimum* L. (0.1%) and O. *kilimandscharicum* L. (1.4%) [25].

### 3.2. Total Food Intake and Total Weight Gain

Total food intake in the BEO-inhaled and linalool-inhaled group was much lower compared to that in the control group (*p* > 0.05) (Table 2); however, there were no significant differences among all groups. In the case of total weight gain, the 20 min BEO-inhaled group showed significant less weight gain compared to the control group (*p* < 0.05); however, the 5 min BEO-inhaled group and the linalool-inhaled group did not show any significant differences compared to the control group.

Basil can modulate body weight, and linalool plays an important role as a ligand of peroxisome proliferator-activated receptor α (PPAR*α*) [7,21]. PPAR*α* can modulate fatty acid uptake and fatty acid oxidation and inhibit the occurrence of obesity. Linalool is commonly used for medicinal functions [21]. This study showed that 20 min of BEO inhalation suppressed total weight gain. In contrast, linalool inhalation did not result in decreased body weight. Previous research reported that BEO induced a decrease in body weight [26] and reduced the average body weight [27]. A linalool-containing essential oil has anti-obesity effects, including decreasing body weight and/or promoting lipolysis [5,28]. In addition, Baek et al. reported that linalool inhibits body weight gain [6]. However, linalool inhalation only suppressed the average body weight gain in this study. Therefore, the reduction in body weight could be due to the complex effects of the aroma components of BEO, rather than the sole effect of linalool.

### 3.3. Forced Swimming Test

Changes in swimming records were measured during the study period (Table 3). During the initial period, no significant differences were observed between the groups. In the final period, the control group had the lowest swimming time among all groups (*p* < 0.05), while the other groups showed increasing swimming time. The BEO-inhaled groups showed increased swimming time in an inhalation time-dependent manner. When comparing the BEO- and the linalool-inhaled groups, the 20 min BEO-inhaled group showed a significant increase in swimming time compared to the control group (*p* < 0.05).

Chronic stress can cause oxidative stress, and animals exposed to oxidative stress have an increased immobility period during forced swimming tests [29,30]. In addition, the period of immobility in rats is decreased by reducing oxidative stress [30]. The results of this study also identified differences between stress-exposed rats and stress-relieved rats following the inhalation of BEO and linalool. A previous study indicated that linalool inhalation upregulated plasma biomarkers and gene expression in rat models of stress [31], while another study indicated that BEO ameliorated oxidative stress in rats [32]. In addition, BEO significantly increased the ambulatory activity via the stimulation of the CNS, and this BEO is considered a potent CNS regulator [33].

### 3.4. Pulse

During the initial period, no significant differences in pulse were observed between the groups (Table 4). In contrast, during the final measurement, inhalation of BEO for 5 min attenuated the pulse rate compared to that in the control group (*p* < 0.05). Inhalation of BEO for 20 min and of linalool only showed a tendency to decrease the pulse, and changes were not significant (*p* > 0.05).

The pulse is controlled by the ANS, which includes sympathetic and parasympathetic nerves, and a reduced pulse is associated with decreased sympathetic and increased parasympathetic nerve activity [4]. In this study, inhalation of BEO and linalool resulted in significantly and/or relatively decreased pulse rates. Previous studies indicated that linalool and linalool-containing essential oils attenuated renal sympathetic nerve activity and enhanced parasympathetic nerve activity by olfactory stimulation, and linalool-containing essential oil inhalation decreased the pulse rates in rats [28,33]. In addition, inhalation of linalool has a sedative effect in animal models [8], and BEO inhalation also induced a sedative effect by decreasing the arousal response measured on the basis of electroencephalographic activity [34].

### 3.5. Organ Weights

The liver, kidney, heart, white adipose tissue (WAT), and brown adipose tissue (BAT) were weighed (Table 5). There were no significant differences in liver weights among the groups. The kidney weights in the 20 min BEO-inhaled group was lower than that of the control group (*p* < 0.05). Meanwhile, inhalation of BEO for 5 min and of linalool induced no significant decrease in liver weight. In terms of heart weight, there were no significant differences between the groups. The control group had the highest WAT weight among all groups. The BEO-inhaled groups showed a decreasing tendency in WAT weights; however, these changes were not significantly different. The linalool-inhaled group showed a decrease in WAT weight compared with the control group (*p* < 0.05). BAT weights were measured in all groups, and there were no significant differences.

WAT is related to oxidative stress, and increased WAT and oxidative stress can increase the metabolic risk [29]. Accumulation of WAT generally increases cardiovascular disorders, being associated with increased levels of TC, LDL, and TG, as well as decreased levels of HDL [17]. This study showed that inhalation of BEO and linalool decreased WAT weight. Linalool inhalation significantly decreased WAT weight compared with the control group (*p* < 0.05). Therefore, the reduction in WAT appeared to occur in a linalool concentration-dependent manner. A previous study reported that linalool reduced WAT weight in mice [6], while another study found that linalool induced lipolysis by upregulating PPAR*α* activity, fatty acid oxidation, and energy metabolism [21]. In addition, linalool treatment significantly reduced lipid accumulation in 3T3-L1 cells [35]. Moreover, research has found decreased fat accumulation following linalool odor stimulation [36].

### 3.6. Stress Hormones

Stress hormones, including cortisol and serotonin, were measured using ELISA kits. Cortisol levels in the control group were the highest among all groups (Table 6). Inhalation of BEO and linalool decreased the cortisol levels; however, these decreases were not statistically significant. The levels of serotonin in the control group were the lowest among all groups. Inhalation of BEO and linalool induced an increase of serotonin; however, these increases showed no significant differences.

### 3.7. Serum Biomarkers and Hepatotoxicity Indicators

Serum biomarkers were measured using a commercial kit. TC in the 20 min BEO-inhaled group was lower than in the control group (*p* < 0.05) (Table 7). However, there were no significant differences when comparing the control, 5 min BEO-inhaled, and linalool-inhaled groups. In the case of HDL levels, the control and linalool-inhaled groups had the lowest levels compared with the BEO-inhaled groups, regardless of the BEO inhalation time (*p* < 0.05). Thus, BEO inhalation upregulated the HDL levels. The control group had the highest LDL levels among all groups (*p* < 0.05). BEO inhalation ameliorated the levels of LDL, and linalool ameliorated the LDL levels compared to the control group (*p* < 0.05). The TG level in the control group was relatively higher than in the other groups (*p* > 0.05). BEO and linalool inhalation were associated with a decreasing tendency of TG levels. Inhalation of BEO for 20 min showed decreased the TG levels compared to the control group (*p* < 0.05). Meanwhile, linalool was associated with a decreasing trend in TG levels compared to the control group; however, there were no significant changes between the control and the linalool-inhaled group. Regarding the atherogenic index (AI) and cardiac risk factors (CRF) in the control group, inhalation of BEO ameliorated the AI and CRF indices in a time-dependent manner (*p* < 0.05). Inhalation of linalool induced no significant effects on AI or CRF. Inhalation of BEO and linalool significantly decreased (*p* < 0.05) the levels of LHR when compared with the control group. In particular, BEO inhalation for 20 min showed the lowest LHR among all the groups (*p* < 0.05). Hepatotoxicity indicators, including AST and ALT, were measured using a commercial kit. When comparing all groups, the AST and ALT levels did not show any significant differences (Table 8). In addition, the AST/ALT ratio was not significantly different among the groups.

Oxidative stress influences the lipid metabolism [37] and usually increases the prevalence of atherosclerosis by increasing reactive oxygen species, nitric oxygen, and oxidized-LDL production and decreasing the levels of antioxidants [38]. LDL is generally associated with the weight of WAT, and increased levels of LDL can promote cardiovascular diseases such as atherosclerosis and dyslipidemia [5]. In contrast, HDL is associated with anti-inflammatory indicators and the presence of antioxidants [5]. Accordingly, HDL plays an important role in cardiovascular health and can reduce the prevalence rate of cardiovascular diseases [39,40]. The results of this study showed that linalool and BEO inhalation decreased the LDL levels in chronically stressed rats. In addition, BEO inhalation ameliorated the levels of HDL in chronically stressed rats, regardless of its concentration. Therefore, BEO and linalool inhalation upregulated the LDL and HDL levels in this study. In particular, the AI and CRF were reduced by BEO inhalation regardless of its concentration (Table 7). A recent study reported that the administration of BEO decreased the levels of TC, LDL, and TG [7]. Similarly, the administration of purple basil essential oil improved hyperlipidemia, lowering triglyceride and total cholesterol levels, similar to our results [31]. Additionally, linalool has cholesterol-lowering, antioxidant, and anti-inflammatory activities [41]. Previous research showed that linalool decreased the LDL levels [6] and activated hepatic PPAR*α* [21]. Therefore, linalool ameliorated dyslipidemia by lowering LDL activity [21]. BEO, a linalool-containing essential oil, has been reported to reduce hyperlipidemia and oxidative stress in rats [31]. Therefore, linalool inhalation played an important role in improving the LDL levels in this study.

The AST and ALT levels are associated with hepatic damage. The AST/ALT ratio is an indicator of liver function impairment [42]. In this study, there were no significant differences in the levels of AST and ALT and in the AST/ALT ratios among all groups. Therefore, inhalation of BEO and linalool had no adverse effects on hepatic and liver function.

## 4. Conclusions

In conclusion, these findings suggest that BEO and linalool inhalation suppresses stress responses, including dyslipidemia. Nevertheless, these findings are limited to animal models of chronic stress. Therefore, further research should be performed to investigate the effects of the inhalation of BEO and linalool in clinical trials. Furthermore, these findings can be of interest in the industry field and suggest the use of odor-active aroma compounds in BEO and linalool to suppress stress, without the intake and/or oral administration of health-promoting compounds.

## Figures and Tables

**Table 1:** Aroma profiles and odor-active aroma compounds in basil essential oil identified using GC–MS and GC–O.

Compounds	Retention Time(min)	Retention Index	Content (ug/mL)	Odor Intensity	Odor Description
Alcohols (5)					
1,8-Cineole	16.29	1061	38.91		
Linalool oxide	17.58	1100	7.33	3	Herb
Linalool	18.37	1128	107.65	4	Sweet, Fruit
Fenchyl alcohol	18.85	1145	32.11		
*trans*-Anethole	23.02	1291	14.39		
Hydrocarbons (10)					
Camphene	13.62	978	5.02		
*β*-Myrcene	14.98	1019	15.72		
*γ*-Terpinene	15.42	1034	11.24		
*α*-Terpinene	15.80	1046	6.22		
*β*-Cymene	16.07	1054	7.77		
D-Limonene	16.20	1058	34.64		
Ocimene	16.44	1066	13.43		
Menthene	20.82	1212	17.33	4	Herb, Basil, Xylitol
2-Hydroxy phenyl butane	24.15	1335	22.29		
1-Methoxy ethyl benzene	24.34	1342	14.65		
Ketones (2)					
Menth-4-en-3-one	22.82	1284	45.40		
D-Carvone	22.89	1287	6.44	1	Lemon

**Table 2:** Total food intake and total weight gain during the animal experiment. CON: chronic stress-exposed control group; POS: linalool (positive control) inhalation by chronic stress-exposed rats; 5 MIN: BEO inhalation for 5 min by chronic stress-exposed rats; 20 MIN: BEO inhalation for 20 min by chronic stress-exposed rats.

	Total Food Intake (g)	Total Weight Gain (g)
CON	499.51 ± 10.60 ^a1^	219.67 ± 2.08 ^a^
POS	483.37 ± 9.73 ^a^	204.67 ± 12.34 ^ab^
5 MIN	467.21 ± 24.37 ^a^	201.67 ± 11.15 ^ab^
20 MIN	479.90 ± 11.72 ^a^	190.67 ± 2.52 ^b^

Data are given as mean ± SD values from experiments performed in triplicate. ^1^ Mean values with different letters within the same row are significantly different according to the non-parametric Friedman test, followed by Dunn’s test (*p* < 0.05).

**Table 3:** Forced swimming test during the initial and final periods. CON: chronic stress-exposed control group; POS: linalool (positive control) inhalation with chronic stress-exposed rats; 5 MIN: BEO inhalation for 5 min by chronic stress-exposed rats; 20 MIN: BEO inhalation for 20 min by chronic stress-exposed rats.

	Initial (s)	Final (s)
CON	71.67 ± 22.19 ^a1^	8.33 ± 5.13 ^b^
POS	44.67 ± 3.51 ^a^	16.67 ± 16.97 ^b^
5-MIN	63.00 ± 6.93 ^a^	18.67 ± 9.87 ^ab^
20-MIN	72.66 ± 10.26 ^a^	47.33 ± 14.84 ^a^

Data are given as mean ± SD values from experiments performed in triplicate. ^1^ Mean values with different letters within the same row are significantly different according to the non-parametric Friedman test, followed by Dunn’s test (*p* < 0.05).

**Table 4:** Pulse assessment using the tail-cuff method in rats.

Pulse (beats/min)	Initial	Final
CON	406 ± 13 ^a1^	420 ± 19 ^a^
POS	407 ± 17 ^a^	416 ± 14 ^a,b^
5 MIN	402 ± 14 ^a^	351 ± 19 ^b^
20 MIN	414 ± 3 ^a^	392 ± 21 ^a,b^

Data are given as mean ± SD values from experiments performed in triplicate. ^1^ Mean values with different letters within the same row are significantly different according to the non-parametric Friedman test, followed by Dunn’s test (*p* < 0.05).

**Table 5:** Changes in rat organ weights.

	Liver (g)	Kidney (g)	Heart (g)	WAT (g)	BAT (g)
CON	11.74 ± 2.87 ^a1^	2.09 ± 0.03 ^a^	1.38 ± 0.03 ^a^	6.86 ± 0.40 ^a^	0.62 ± 0.06 ^a^
POS	8.83 ± 0.19 ^a^	1.97 ± 0.02 ^a,b^	1.33 ± 0.11 ^a^	5.75 ± 0.61 ^b^	0.71 ± 0.10 ^a^
5 MIN	9.00 ± 0.28 ^a^	2.11 ± 0.05 ^a^	1.30 ± 0.29 ^a^	6.46 ± 0.29 ^a,b^	0.68 ± 0.05 ^a^
20 MIN	9.96 ± 0.29 ^a^	1.86 ± 0.03 ^b^	1.38 ± 0.08 ^a^	6.15 ± 0.16 ^a,b^	0.59 ± 0.03 ^a^

Data are given as mean ± SD values from experiments performed in triplicate. ^1^ Mean values with different letters within the same row are significantly different according to the non-parametric Friedman test, followed by Dunn’s test (*p* < 0.05).

**Table 6:** Changes in stress hormones in rats.

	Cortisol (ng/mL)	Serotonin (ng/mL)
CON	25.34 ± 1.12 ^a1^	8.25 ± 0.90 ^a^
POS	20.99 ± 8.96 ^a^	9.72 ± 0.38 ^a^
5 MIN	24.77 ± 3.14 ^a^	9.09 ± 1.01 ^a^
20 MIN	24.16 ± 4.24 ^a^	9.34 ± 0.86 ^a^

Data are given as mean ± SD values from experiments performed in triplicate. ^1^ Mean values with different letters within the same row are significantly different according to the non-parametric Friedman test, followed by Dunn’s test (*p* < 0.05).

**Table 7:** Effects of basil essential oil and linalool inhalation on lipid metabolism in chronically stressed rats. CON: chronic stress-exposed control group; POS: linalool (positive control) inhalation by chronic stress-exposed rats; 5 MIN: BEO inhalation for 5 min by chronic stress-exposed rats; 20 MIN: BEO inhalation for 20 min by chronic stress-exposed rats.

	TC (mg/dL)	HDL (mg/dL)	LDL (mg/dL)	TG (mg/dL)	AI	CRF	LHR (mg/dL)
CON	131.23 ± 6.29 ^a1^	46.79 ± 2.83 ^b^	84.43 ± 3.47 ^a^	46.78 ± 5.33 ^a^	1.81 ± 0.26 ^a^	2.81 ± 0.26 ^a^	180.71 ± 9.05 ^a^
POS	121.62 ± 4.43 ^ab^	47.17 ± 2.88 ^b^	73.78 ± 1.51 ^b^	40.81 ± 3.10 ^ab^	1.58 ± 0.07 ^ab^	2.58 ± 0.07 ^ab^	156.84 ± 11.28 ^b^
5 MIN	131.10 ± 3.28 ^a^	55.34 ± 1.60 ^a^	75.76 ± 1.97 ^b^	43.32 ± 1.54 ^a^	1.37 ± 0.05 ^b^	2.37 ± 0.05 ^b^	136.91 ± 1.76 ^b^
20 MIN	110.30 ± 0.59 ^b^	56.46 ± 2.70 ^a^	53.94 ± 2.72 ^c^	34.36 ± 0.17 ^b^	0.97 ± 0.10 ^c^	1.97 ± 0.10 ^c^	95.83 ± 9.36 ^c^

Data are given as mean ± SD values from experiments performed in triplicate. ^1^ Mean values with different letters within the same row are significantly different according to the non-parametric Friedman test, followed by Dunn’s test (*p* < 0.05).

**Table 8:** Effects of basil essential oil and linalool inhalation on hepatotoxicity in chronically stressed rats. CON: chronic stress-exposed control group; POS: linalool (positive control) inhalation with chronic stress-exposed rats; 5 MIN: BEO inhalation for 5 min b chronic stress-exposed rats; 20 MIN: BEO inhalation for 20 min by chronic stress-exposed rats.

	AST (Karmen/mL)	ALT (Karmen/mL)	AST/ALT
CON	153.16 ± 1.82 ^a1^	25.24 ± 0.07 ^a^	6.07 ± 0.09 ^a^
POS	155.65 ± 2.67 ^a^	24.84 ± 0.58 ^a^	6.27 ± 0.23 ^a^
5 MIN	151.94 ± 1.43 ^a^	24.05 ± 0.55 ^a^	6.18 ± 0.03 ^a^
20 MIN	151.34 ± 1.89 ^a^	24.58 ± 0.12 ^a^	6.29 ± 0.14 ^a^

Data are given as mean ± SD values from experiments performed in triplicate. ^1^ Mean values with different letters within the same row are significantly different according to the non-parametric Friedman test, followed by Dunn’s test (*p* < 0.05).

## Data Availability

The data presented in this study are available.
